# The complete mitochondrial genome of *Heliocidaris crassispina* (A. Agassiz, 1864)

**DOI:** 10.1080/23802359.2019.1674712

**Published:** 2019-10-11

**Authors:** Wenfei Zhao, Zhe Li, Xiaofang Huang, Yang Zhang, Yaqing Chang, Jun Ding

**Affiliations:** Key Laboratory of Mariculture and Stock Enhancement in North China's Sea, Ministry of Agriculture and Rural Affairs, Dalian Ocean University, Dalian, P. R. China

**Keywords:** Mitochondrial genome, sea urchins, *Heliocidaris crassispina*

## Abstract

In this study, the complete mitochondrial genome of *Heliocidaris crassispina* was determined on Illumina HiSeq platform. The genome was 15,678 bp in size and contains 22 tRNA genes, 13 protein-coding genes, 2 rRNA genes and 1 control region (96 bp). The composition of A + T in *H. crassispina* mtDNA was 59.11%. Except ND6 and 5 tRNAs, the others are on the H-strand. The phylogenetic relationship of 11 species of sea urchins was analyzed using the neighbour-joining method by software MEGA 7.0. *H. crassispina* was most closely related to *Pseudocentrotus depressus.*

*Heliocidaris crassispina* belong to the Family of Echinometridae. *H. crassispina* lived in warm water and narrow salt species. It is suitable for growing at 15–30° C and 25–30 salinity. It usually lives in shallow water areas of low-tide and subtidal zones along the coast of southern China (Freeman 2003). By comparing with the mitochondrial genome of other sea urchins, it provides basic data and important scientific data for the phylogenetic evolution and diversity of sea urchins. Therefore, the research on the mitochondrial whole genome of *H. crassispina* becomes very important.

In the study, our sample was collected from Zhoushan sea area, Zhejiang province, China (30°11'N 122°41'E). The samples and its DNA were stored at −80 °C before sequencing. The specimen is stored in the Key Laboratory of Mariculture and Stock Enhancement in North China's Sea, Ministry of Agriculture and Rural Affairs, Dalian Ocean University (voucher number: DLOU-KLM-SU06). The complete mitochondrial genome of *H. crassispina* was determined on Illumina HiSeq platform. Using SOAP denovo V2.04 software (http://soap.genomics.org.cn/) to assemble sequence (Luo et al. 2012). DOGMA software (http://dogma.ccbb.utexas.edu/) was used to predict gene, rRNA, and tRNA contained in the genome. MEGA7.0 (Kumar et al. 2016) was used for multiple alignments. The mtDNA map of *H. crassispina* mitochondrial genome was drawn with the online tool OGDraw (https://chlorobox.mpimp-golm.mpg.de/OGDraw.html) (Lohse et al. 2013).

The mitochondrial genome of *H. crassispina* has a total length of 15,678 bp (GenBank registration number: MN275902), including 13 protein-coding genes, 22 tRNA genes, 2 rRNA genes and a non-coding region with a length of 96 bp. The mitochondrial genome of *H. crassispina* g composition is 31.14% for A, 24.28% for C, 16.61% for G and 27.69% for T. The composition of A + T in *H. crassispina* mtDNA was 59.10%. Except ND6 and 5 tRNAs (tRNA-Asp, tRNA-Val, tRNA-Ala, tRNA-Gln, tRNA-Ser), the others are on the H-strand. All protein initiation codons are ATG, except for ATP8. The termination codon of most protein-coding genes was TAG (8 of 13 genes) or TAA (3 of 13 genes). The size of 22 tRNA coding genes ranged from 68 bp to 73 bp.

The phylogenetic relationship of 12 species of sea urchins was analyzed using the neighbour-joining method by MEGA 7.0. The results showed that the *H. crassispina* were most closely related to the *Pseudocentrotus depressus* (Figure 1). The result can contribute to construct molecular identification of this species and be helpful to explore the phylogeny of Echinometridae.

**Figure 1.**
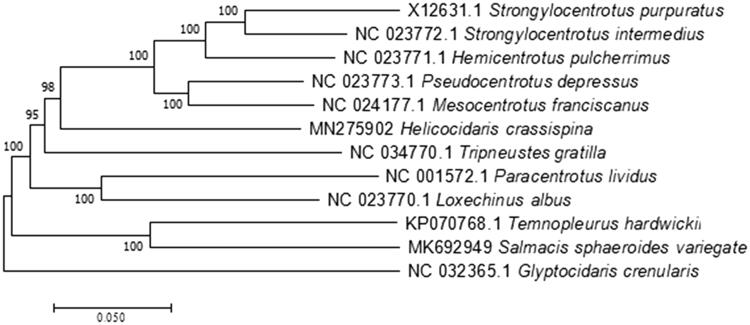
Consensus neighbour-joining tree based on the complete mitochondrial sequence of *H. crassispina* and other 12 species of sea urchins. The phylogenetic tree was constructed using MEGA 5.0 software by the neighbour-joining method. The numbers at the tree nodes indicates the percentage of bootstrapping after 1000 replicates.
